# Cyanobacterial Xenobiotics as Evaluated by a *Caenorhabditis elegans* Neurotoxicity Screening Test

**DOI:** 10.3390/ijerph110504589

**Published:** 2014-04-25

**Authors:** Jingjuan Ju, Nadine Saul, Cindy Kochan, Anke Putschew, Yuepu Pu, Lihong Yin, Christian E. W. Steinberg

**Affiliations:** 1 Key Laboratory of Environmental Medicine Engineering, Ministry of Education, School of Public Health, Southeast University, Nanjing 210009, China; E-Mails: jujingjuan@gmail.com (J.J.); yppu@seu.edu.cn (Y.P.); 2 Department of Biology, Freshwater and Stress Ecology, Humboldt-Universität zu Berlin, Späthstr. 80/81, Berlin 12437, Germany; E-Mail: nadines1976@aol.com; 3 Chair of Water Quality Control, Technische Universität Berlin, Straße des 17. Juni 135, Berlin 10623, Germany; E-Mails: cindy.kochan@tu-berlin.de (C.K.); anke.putschew@tu-berlin.de (A.K.)

**Keywords:** *C. elegans*, neurotoxicity, automatic function, sensory function, anatoxin-*a*, microcystin-LR

## Abstract

In fresh waters cyanobacterial blooms can produce a variety of toxins, such as microcystin variants (MCs) and anatoxin-*a* (ANA). ANA is a well-known neurotoxin, whereas MCs are hepatotoxic and, to a lesser degree, also neurotoxic. Neurotoxicity applies especially to invertebrates lacking livers. Current standardized neurotoxicity screening methods use rats or mice. However, in order to minimize vertebrate animal experiments as well as experimental time and effort, many investigators have proposed the nematode *Caenorhabditis elegans* as an appropriate invertebrate model. Therefore, four known neurotoxic compounds (positive compounds: chlorpyrifos, abamectin, atropine, and acrylamide) were chosen to verify the expected impacts on autonomic (locomotion, feeding, defecation) and sensory (thermal, chemical, and mechanical sensory perception) functions in *C. elegans*. This study is another step towards successfully establishing *C. elegans* as an alternative neurotoxicity model. By using this protocol, anatoxin-*a* adversely affected locomotive behavior and pharyngeal pumping frequency and, most strongly, chemotactic and thermotactic behavior, whereas MC-LR impacted locomotion, pumping, and mechanical behavior, but not chemical sensory behavior. Environmental samples can also be screened in this simple and fast way for neurotoxic characteristics. The filtrate of a *Microcystis aeruginosa* culture, known for its hepatotoxicity, also displayed mild neurotoxicity (modulated short-term thermotaxis). These results show the suitability of this assay for environmental cyanotoxin-containing samples.

## 1. Introduction

The worldwide proliferation of cyanobacterial blooms, most often dominated by *Microcystis* sp., is caused by eutrophication of water bodies and increases in temperature due to global climate change [1]. The *Microcystis* sp. is well known for producing a great variety of toxic peptides, such as aeruginosins, microginins, anabaenopeptins, cyanopeptolins, microviridins, and particularly microcystins [2]. For microcystins (MCs) the liver is the main target and they are therefore classified as hepatotoxins [3], however neurotoxic side-effects have also been observed for a long time. For instance, during the classical case of MC-intoxication in a Brazilian hemodialysis unit, even neurotoxic symptoms were observed [4]. Furthermore, it has to be taken into consideration that MC-producing cyanobacterial blooms are frequently contaminated or mixed with cyanobacteria producing additional toxins, particularly with those producing neurotoxic compounds, such as anatoxin-*a* (ANA) [5]. Probably even more important than these contaminations, strains of the dominant bloom-forming cyanobacterial genus, *Microcystis*, are capable of producing ANA themselves, even in relatively high concentrations [6]. All of these studies indicate that in blooms of *Microcystis*, not only hepatotoxic but even neurotoxic potentials should be expected. Recent hole-genomic DNA microarray studies demonstrate that in liver-lacking invertebrates neurotoxicity appears to be the major intoxication pathway even for MC-LR [7]. So far, however, this potentially important toxicity has gained little attention. 

Neurotoxic effects serve as important endpoints in environmental toxicity analyses in general. Currently established guidelines are available only for mammalian models [8]. However, these tests are complex, expensive, and time consuming. Additionally, ethical issues limit the use of these tests and underline the need for alternative and/or additional, simple, but robust methods. The nematode *C. elegans*, as an emerging model in environmental toxicology [9], offers several advantages: it is easy and inexpensive to culture in the laboratory and its short life cycle allows for completion of the procedure within a short time span. However, the most interesting characteristic of *C. elegans* is that increasing evidence shows its similarity with mammals, both on the genetic and the physiological level [10]. Thus, the results from *C. elegans* have the potential to predict possible effects in higher animals. 

Alterations in the behavior of C. *elegans* may reflect changes at the cellular or functional level. Therefore, behavioral assays offer a simple, sensitive, and powerful tool to explore the neurotoxicity of environmental substances. *C. elegans* has served as a successful model for assessing selected neurotoxic chemicals by using various behavioral endpoints [11,12,13,14,15,16,17,18]. The behavioral endpoints of *C. elegans* could even be used to distinguish neurotoxic mammalian chemicals from non-neurotoxic chemicals, such as organophosphates, metals and organic solvents [12]. In general, exposure of *C. elegans* to chemicals including pesticides and metals resulted in a reduction of behavioral frequency at exposure concentrations below lethality levels [19,20]. Using these behavioral endpoints of *C. elegans* to assess the toxicity of chemicals not only yields high sensitivity but also consistency with prior results in mammalian models [13,20,21]. Therefore, we tried to validate the *C. elegans* model with well-known neurotoxic substances by using systematic behavioral endpoints, which were selected according to the established mammalian guidelines. In addition, one *Microcystis aeruginosa* culture filtrate (*Ma*cf) containing a mixture of cyanotoxins [22] as well as the neurotoxin anatoxin-*a* (ANA) and the microcystin-LR (MC-LR) were then assessed by this model. Although the major mode of toxic action of MCs is hepatotoxicity, MCs were also shown to be generally toxic [23] and even neurotoxic for the liver-lacking *C. elegans*. These neurotoxic effects of MCs were shown with some scattered phenotypic endpoints [24,25,26] and on the biomolecular level [7], but not yet in a systematic fashion with cyanotoxins and easy-to-handle endpoints. Overall, neurotoxic responses of *C. elegans* to the positive compounds, and to *Ma*cf as well as the two cyanotoxins, would prove the general suitability of the test system for environmental samples.

## 2. Experimental Section

### 2.1. Strains

The wild-type *C. elegans* strain N2 and the *Escherichia coli* strain OP50 were obtained from the Caenorhabditis Genetics Center (Minneapolis, MN USA). L4 larvae were used as the starting exposure stage. Nematodes were maintained on nematode growth medium (NGM) plates seeded with OP50 at 20 °C as described by Brenner *et al.* [27]. All quantitative measurements were carried out using a digital microscope (Keyence VHX 600D, Osaka, Japan) or a stereo microscope (Nikon, Tokyo, Japan).

### 2.2. Preparation of Plates and Exposure Conditions

MC-LR and ANA (Enzo Life Sciences, Lörrach, Germany) were dissolved by DMSO and then added to the NGM agar and the OP50 bacteria with final concentrations ranging from 0.1 to 100 µg·L^‑1^. The final concentration of the solvent DMSO was 0.3% (v/v).

The positive neurotoxic compounds which include chlorpyrifos (Sigma-Aldrich, Steinheim, Germany) for the locomotion assay, abamectin (Sigma-Aldrich) for the pharyngeal pumping assay, atropine (Sigma-Aldrich) for the defecation assay, and acrylamide (Sigma-Aldrich) for sensory function were prepared as mentioned above.

To check the applicability of the neurotoxicity assay with an aquatic sample, a filtrate was taken from a *M. aeruginosa* (Culture Collections of Algae at the University of Göttingen, SAG, Göttingen, Germany) batch culture grown in 20 L pure Z-medium amended with 200 µg·L^−1^ bromide. The OP50 bacteria as well as the NGM medium were spiked with 40% of this filtrate, resulting in a final total MC concentration of about 300 µg·L^−1^ consisting of three variants as given in Table 1.

**Table 1:** Contents of microcystins from the culture filtrate of *Microcystis aeruginosa.*

The Culture Filtrate	Microcystins, µg·L^−1^
*Microcystis aeruginosa* batch culture	MC-RR 244 ± 7%
in Z-medium + 200 µg·L^−1^·Br	MC-YR 180 ± 11%
	MC-LR 332 ± 3%

Each neurotoxic test was displayed after exposing the nematodes for 24 and 72 h. At least two replicates and 8–30 nematodes per replicate were used in each assay. Locomotion, pharyngeal pumping and the defecation assay were taken as an assay of autonomic function, while sensory function was assessed by chemotaxis, thermotaxis and mechanical assay.

### 2.3. Analysis of Microcystins in the Culture Filtrate

According to DIN 20179 solid phase extraction (SPE) was used to concentrate dissolved Microcystins from water samples prior to HPLC. Five hundred mL of water was passed through Lichrolute RP 18 (300 mg, 3 cc, Merck Millipore, Darmstadt, Germany) SPE cartridges, conditioned with 4 mL 80% methanol and eluted with 6.5 mL methanol that was acidified with 0.1% formic acid (v/v). The organic solvent was removed with a gentle nitrogen stream. Extracts were dissolved in 0.5 mL ultrapure water and analyzed by an LC system with Triple-Quad MS TSQ Vantage (Thermo Fisher Scientific, Waltham, MA, USA) using an electrospray (ESI) interface. Separation was achieved on a Zorbax Eclipse XDB C18-UPLC column at 40 °C with a flow rate of 0.4 mL·min^−1^. For LC/MS-MS gradient elution both water (A) and methanol (B) contained 0.006% acetic acid (v/v) and 5 mM HCOONH_4_. The gradient for microcystin separation was 0%–2% B, 0–1.5 min; 2%–50% B, 1.5–5 min; 50%–70% B, 5–8 min; 70% B, 8–13 min; 0% B for re-equilibration. The ESI+ multiple reaction monitoring transitions were m/z 519.8 → 135.0 and 239.1 (Microcystin-RR), m/z 995.6 → 135.0 and 861.0 (Microcystin-LR) and 1045.5 → 135 and 174.0 (Microcystin-YR). The limit of quantification was 10 ng·L^−1 ^for each microcystin. Further details are given in Ju *et al.* [28].

MC-RR and MC-YR standards were obtained from Enzo Life Science (solid substances, purity 95%). All solvents used were HPLC grade. The ultrapure water was produced through deionization with a water purification system (maxima ultrapure water; Elga, Upstadt-Weiher, Germany). The cyanotoxins were dissolved in methanol to reach a concentration of 9.5 mg·L^−1^ as a stock solution.

### 2.4. Locomotion Behavior

After exposing *C. elegans* for 24 or 72 h, body bend frequency and relative move length were determined to monitor the locomotive behavior. A body bend was counted as a change in the direction of *C. elegans* movement. To determine the relative move length, nematodes were transferred to fresh plates for 20 s. After removal, the crawler lanes in the OP50 lawn were measured. The body length was used for normalization. Twenty worms per treatment were examined for each replicate using a digital microscope.

### 2.5. Pharyngeal Pumping

The pharyngeal pumping rate represents food intake. It was quantified after exposing the nematodes to the positive chemicals as well as cyanobacterial material for 24 or 72 h. Nematodes of the different exposure groups were randomly selected and their pumping frequency was determined three times over a 60 s timespan. Twenty worms per treatment were examined for each replicate using a digital microscope.

### 2.6. Defecation Assay

**Figure 1:**
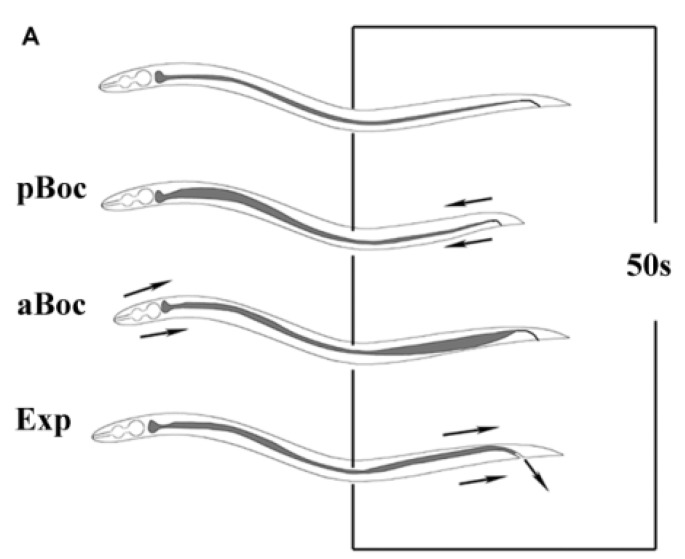
Defecation steps in *C. elegans*.

Notes: pBoc = posterior body-wall muscle contraction; aBoc = anterior body-wall muscle contraction; Exp = Expulsion.

Defecation was assayed as previously described [29]. To a high degree of accuracy, the posterior body-wall muscle contraction of defecation can be clearly recognized. The whole defecation process includes four steps (Figure 1) and we measured the interval between the two posterior body-wall muscle contractions. Twenty worms per treatment were examined for each of the replicates using a digital microscope.

### 2.7. Chemotaxis Assay

Chemotaxis assays were conducted following the protocol described by Saeki, Yamamoto, and Iino [30]. This assay is based on the *C. elegans*’ attraction to NaCl. If impaired, the chemotaxis of the nematodes towards NaCl decreases dramatically. Assay plates (5 mM potassium phosphate, pH 6.0, 1 mM CaCl_2_, 1 mM MgSO_4_ and 20 g·L^−1^ agar) were prepared in advance.

**Figure 2:**
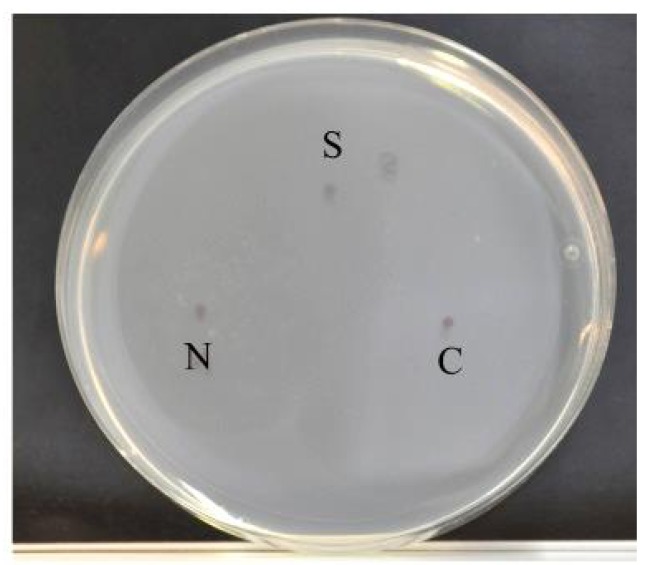
Assay plates for chemotaxis

Notes: S: Start point; N: NaCl + NaN_3_; C: NaN_3_.

An agar plug, which was prepared as described above, but with the addition of 100 mM NaCl, was excised from a NaCl plate and placed on the off-center spot (N, Figure 2) of the assay plate. This plate was then left overnight (14–24 h). Immediately prior to the assay, the NaCl plug was removed and 1 μL sodium azide (0.5 M) was spotted onto the same position to anaesthetize the animals. As a control, the same volume of sodium azide was also spotted onto a position (C, Figure 2) about 4 cm from the NaCl spot. Thirty worms were placed on the starting spot (S, Figure 2), which was about 3 cm away from the C and N spots, and were left to move freely on the assay plate for 1 h at 20 °C. After this amount of time, the number of worms around each spot was counted. The chemotaxis index (CI) was calculated as CI = (N_N_−N_C_)/30, where N_N_ is the number of animals within 1.5 cm of the center of the NaCl gradient and N_C_ is the number within 1.5 cm of the control spot. Assays were performed in triplicate, and the CI value was averaged. Thirty worms per treatment were assayed for each of the replicates using a stereo microscope.

### 2.8. Thermotaxis Assay

**Figure 3:**
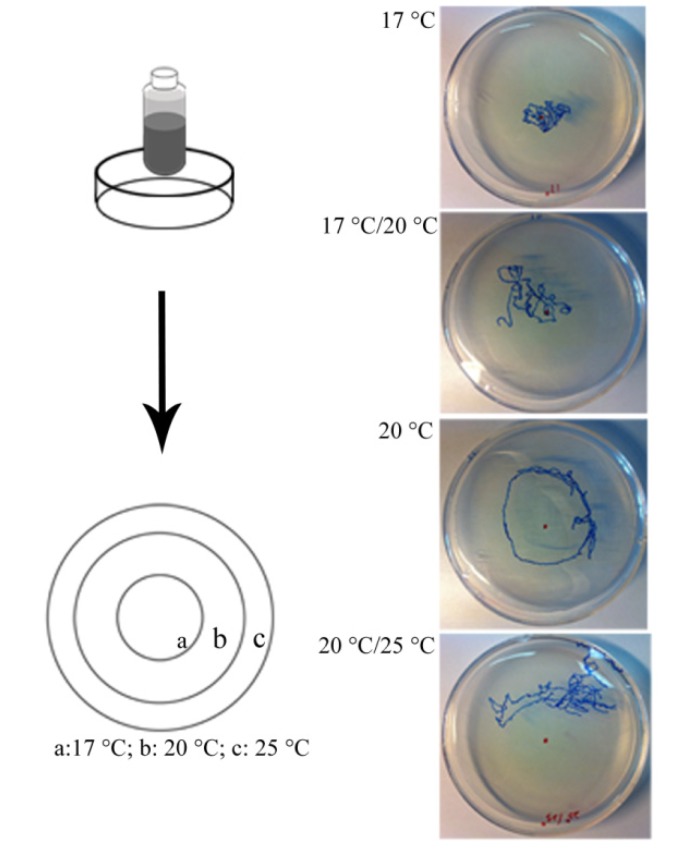
The thermal-gradient assay was performed using a 9 cm plate and a vial containing frozen acetic acid (**Left**); Traces of individuals of each category after 1 h (**Right**).

The thermotaxis assay was performed as described by Kuhara *et al.* [31]. Individual *C. elegans* “memorize” temperatures, and this stored information modifies their subsequent migration along a temperature gradient. Control and exposed nematodes were kept at 20 °C for 24 h or 72 h on a fresh OP50 lawn. Individual nematodes were then transferred onto a fresh plate (9 cm diameter) without bacteria and cultivated at 25 °C. These plates are characterized by a pre-existing radial temperature gradient (Figure 3). The nematodes were placed on the 20 °C area and allowed to move freely on the plates. After 1 h, the trace was analyzed. If the animals moved within distinct temperature areas, their tracks were classified as “17 °C”, “20 °C”, or “25 °C”. Animals that moved between two distinct areas were classified as “17 °C/20 °C” or “20 °C/25 °C”. Between eight and 15 worms per treatment and replicate were assayed using a stereo microscope.

### 2.9. Mechanical Sensory Stimulus

Nose touch response, which is an expression of mechanical sensory perception, was conducted as previously described [32]. The tip of the nose of a forward-moving animal was touched with a fine hair, and reversal was scored as a response. Twenty worms per treatment were assayed for each of the replicates using a stereo microscope.

## 3. Data Analysis

All data are displayed as the mean ± Standard Error of the Mean (SEM). A student’s *t*-tests or ANOVA were used to determine the statistical significance between control and exposed groups. This was followed by the Fisher’s Least Significant Difference (LSD) test or the Dunnett’s *t*-test to determine the significance of differences between the groups. Probability levels of 0.05 and 0.01 were considered statistically significant.

## 4. Results

### 4.1. Autonomic Function

**Figure 4:**
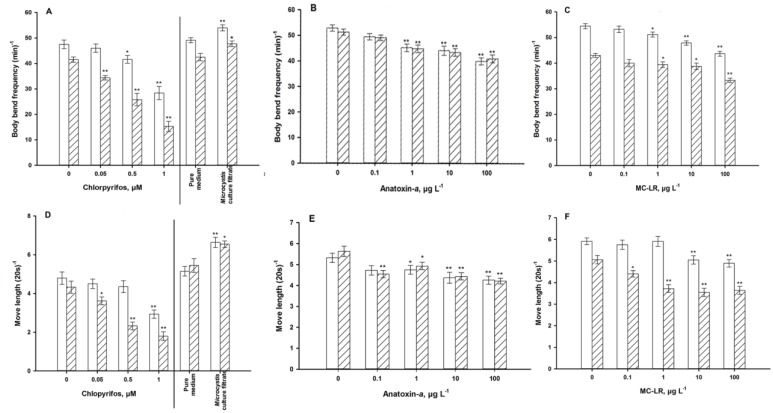
Locomotive behavior of young adult nematodes for 24 h (**white**) and 72 h (**hatched**). Move length is normalized by the mean of body length per group. (**A,D**): exposure to chlorpyrifos and a *Microcystis* culture filtrate. (**B,E**): exposure to anatoxin-*a*. (**C,F**): exposure to microcystin-LR. * = *p* < 0.05, ** = *p* < 0.01.

A gradual and time-related decrease in body bend frequency and move length was evident as chlorpyrifos-exposure increased (Figure 4A,D). After 72 h, even 0.05 μM caused a decrease in body bend frequency. The movement speed, measured as body-length–normalized move length in 20 second, showed a noticeable decrease upon exposure to 1.0 μM chlorpyrifos after 24 h and to 0.5 and 1.0 μM after 72 h.

**Figure 5:**
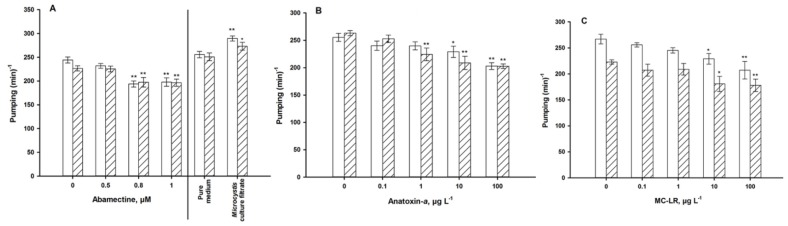
Pharyngeal pumping of young adults for 24 h (**white**) and 72 h (**hatched**). (**A**): exposure to abamectine, and a *Microcystis* culture filtrate. (**B**): exposure to anatoxin-*a*. (**C**): exposure to microcystin-LR. * = *p* < 0.05, ** = *p* < 0.01.

**Figure 6:**
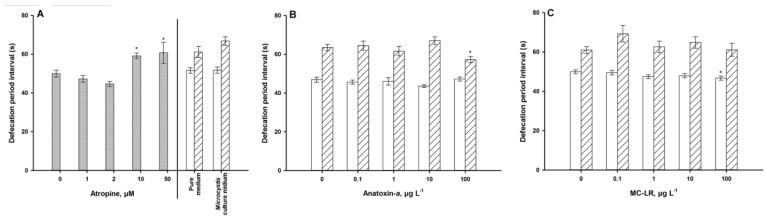
Defecation of young adults. (**A**) exposure to atropine only for 1 h (**grey**) and a *Microcystis* culture filtrate for 24 h (**white**) and 72 h (**hatched**). (**B**): exposure to anatoxin-*a*for 24 h (**white**) and 72 h (**hatched**). (**C**): exposure to microcystin-LR for 24 h (**white**) and 72 h (**hatched**). * = *p* < 0.05, ** = *p* < 0.01.

Exposure to abamectin for 24 h and 72 h resulted in a decline of the mean pharyngeal pumping rate; especially for the exposure concentrations of 0.8 and 1 μM which showed a significant decrease in pumping compared with the control (Figure 5A). Due to its low persistence [33], the atropine exposure trials were terminated after 1 h. The whole defecation process, which is important for measuring atropine response, includes four steps (Figure 1). After exposure to more than 10 µM of atropine for 1 h, the defecation periodicity was significantly prolonged (Figure 6A). However, no worm constipation was observed.

As a well-known neurotoxin, ANA not surprisingly adversely affected both of the locomotive behavior variables (Figure 4B,E) and the pharyngeal pumping frequency (Figure 5B), with a defecation activity of only 100 µg·L^−1^ after 72 h of exposure (Figure 6B). Older worms need longer defecation periods, however, this activity was not modulated by ANA.

The hepatotoxin MC-LR, too, reduced body bends, move length (Figure 4C,F) and pharyngeal pumping frequency (Figure 5C), but not defecation activity (Figure 6C). Exposure to *Ma*cf significantly increased the motile activity (Figure 4A,D), pumping activity (Figure 5A), and after long-term exposure tended to increase the defecation period (Figure 6A).

### 4.2. Sensory Function

**Figure 7:**
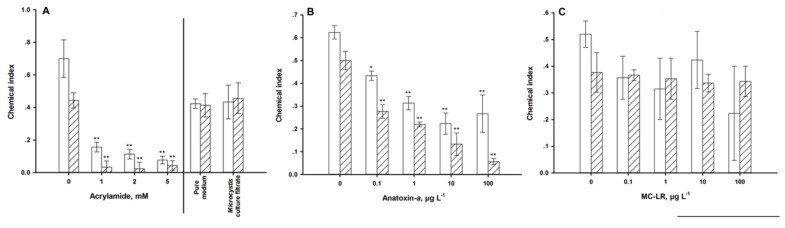
Chemical sensory response of young adult nematodes after exposure for 24 h (**white**) and 72 h (**hatched**). The smaller the chemical index, the worse the chemotactic behavior. (**A**): exposure to acrylamide and a *Microcystis* culture filtrate. (**B**): exposure to anatoxin-*a*. (**C**): exposure to microcystin-LR. * = *p* < 0.05, ** = *p* < 0.01.

When *C. elegans* was fed on NGM (with NaCl and food), its chemotaxis index was around 0.5 after 24 h and 0.4 after 72 h of exposure. The chemotaxis towards NaCl fell dramatically if *C. elegans* was exposed to acrylamide (Figure 7A). 

**Figure 8:**
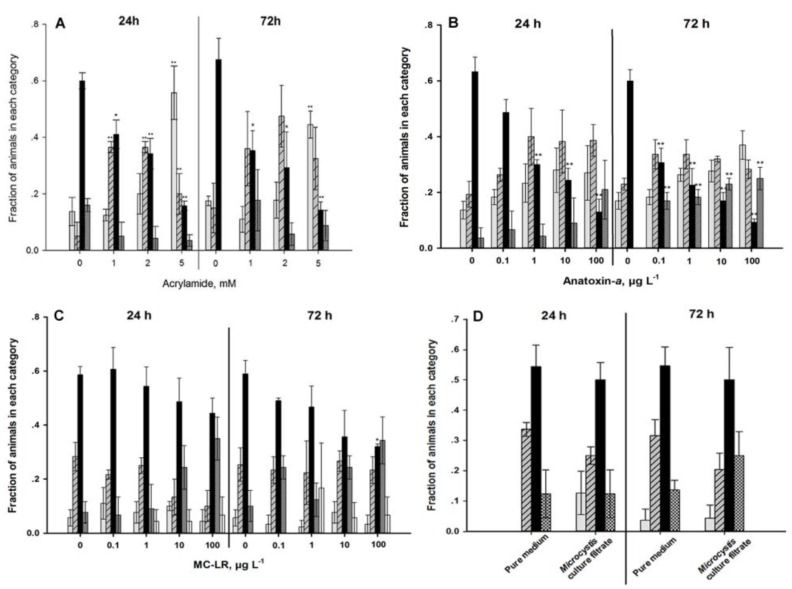
Thermotaxis of young adult nematodes after exposure for 24 h and 72 h. (**A**) exposure to acrylamide and a Microcystis culture filtrate. (**B**): exposure to anatoxin-a. (**C**): exposure to microcystin-LR. (**D**) exposure to a *Microcystis* culture filtrate. * = *p* < 0.05, ** = *p* < 0.01. (**grey**: 17 °C, **hatched**: 17/20 °C, **black:** 20 °C, **dark grey:** 20/25 °C, **white:** 25 °C).

The thermotaxis was also significantly changed. As the exposure concentration of acrylamide increased, the fractions of worms which migrated towards 20 °C decreased, and the worms became more attracted by lower temperatures (Figure 8A).

**Figure 9:**
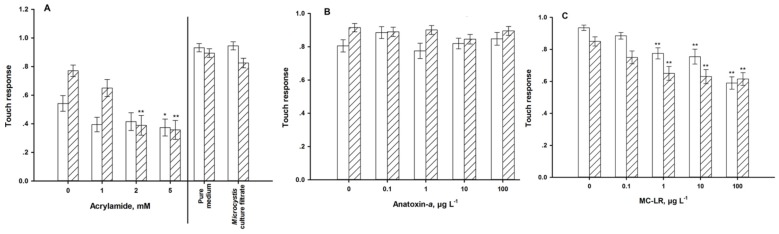
Mechnical sensory function of young adults for 24 h (**white**) and 72 h (**hatched**). (**A**): exposure to acrylamide and a *Microcystis* culture filtrate. (**B**): exposure to anatoxin-*a*. (**C**): exposure to microcystin-LR. * = *p* < 0.05, ** = *p* < 0.01.

The nose touch avoidance behavior was affected by exposure to acrylamide (Figure 9A). Worms exposed to 5 mM of acrylamide for 24 h exhibited a significant decrease in their nose touch avoidance rate. When the exposure duration was increased to 72 h, the worms displayed a significant decrease, even at 2 mM.

The strongest impacts of ANA on sensory function were found in the chemotactic (Figure 7B) and the thermotactic behaviors (Figure 8B). Exposed worms were increasingly not attracted to NaCl and preferred colder and warmer temperatures. Long-term exposure aggravated these effects. The mechanical sensory behavior remained unaffected by ANA exposure (Figure 9B).

In contrast to ANA, MC-LR impacted the mechanical (Figure 9C), but not the chemical sensory behavior (Figure 7C). MC-LR-exposure also changed the thermotactic behavior; but again unlike the effects of ANA, it caused the nematodes to prefer warmer environments (Figure 8C). Exposure to ≥1 µg·L^−1^ MC-LR forced between 5 and 18% of the individuals to migrate to the 25 °C environment.

*Ma*cf-exposure did not modulate the chemical or mechanical sensory functions (Figure 7A and Figure 9A), but temporarily impacted the thermotactic behavior (Figure 8D). Untreated *C. elegans* migrated to its growth temperature (20 °C) and then moved isothermally [34]. Exposure to *Ma*cf for 24 h reduced the fractions of worms which tended towards 20 °C, the worms were instead more attracted by lower temperatures (Figure 8D). This behavior was no longer observed after 72 h of exposure. *Ma*cf contained three MC variants (Table 1).

## 5. Discussion

### 5.1. Applicability of C. elegans as a Model for Neurotoxicity

The changes in the autonomic and sensory functions of *C. elegans* took place after exposure to chemicals well identified as neurotoxic compounds by tests in mammals. The autonomic function (locomotion behavior, pharyngeal pumping, and defecation) was significantly affected by chlorpyrifos, abamectin, and atropine. The organophosphate insecticide chlorpyrifos acts on the nervous system by inhibiting acetylcholinesterases; it decreases the locomotor activity of mammalians and other species as well as *C. elegans* [35]. The pesticide abamectin was reported to elicit a potent and persistent paralysis of the nematode’s pharynx [36]. Atropine is known to reduce secretions and is used as a drug for functional gastrointestinal disorders [37] and, indeed, we observed prolonged defecation periods upon exposure. These three chemicals showed the expected effect in *C. elegans*. 

Acrylamide acts primarily on nerve terminals and causes ataxia, skeletal muscle weakness and numbness in the hands and feet of animals and humans [38]. It also affects the life span and the expression of genes encoding detoxification enzymes in *C. elegans* [39] and showed the expected effects on sensory function in the assays. Hence, *C. elegans* responded to all positive neurotoxic compounds. 

Consequently, a chemical or a mixture of chemicals may be classified as neurotoxic, if one life trait of exposed *C. elegans* is responsive. Obviously, *C. elegans* can only be a valuable toxicity model if its results are predictive in higher organisms. In addition to several existing comparative studies on the ranking of chemicals between *C. elegans* and mammals, which mainly concentrated on lethality in AchE inhibition and movement [13,20,21], our work supported the consistency of several neurotoxic compounds on various behavioral endpoints. The similarity of the mechanisms should be observed in further study. Another next step in establishing *C. elegans* as a suitable invertebrate replacement for neurotoxic evaluation will be to perform a cross-check with the neurotoxic compounds. For instance, it is of interest whether or not chlorpyrifos and atropine impair sensory function or if acrylamide impairs autonomic or cognitive functions in mammals. After the eventual successful establishment of these effects, this model could save resources and improve efficiency by serving as a prescreening test before mammalian tests.

### 5.2. Cyanotoxins and M. aeruginosa Culture Filtrate

Nowadays, we have quantitative ELISA, HPLC methods and mass spectroscopic methods which allow for the detection and quantification of all known classes of cyanobacterial toxins. However, these tests can only prove the existence of the toxins they cannot show their effects since toxicity is not an intrinsic property of the xenobiotic chemical itself, but rather is displayed only by its interaction with exposed/administered individuals. Consequently, biotests can fill this gap.

The concentrations of the cyanotoxins applied in this study lie below the maximal values found in eutrophicated freshwater bodies [5] and cover well the range of values reported from German lakes and reservoirs [40,41]. The major toxic mode of action of MC-LR is hepatotoxicity and of ANA neurotoxicity [42]. In the *C. elegans* assay which was applied, the neurotoxicity of ANA was well reflected by five out of six test variables indicating the robustness of the applied assays. Particularly, chemotaxis was strongly impaired, deteriorating with exposure time. This indicates that *C. elegans* loses its ability to avoid adverse environments. Translating this finding to natural environments, it reflects the risk of extinction of the population, because a persistence of the population in chemically hostile environments cannot be warranted, if the nematode can no longer avoid them.

For a long time, MCs were considered only hepatotoxic [3,43]. A classic case of severe MC-intoxication, however, which happened in a hemodialysis unit in Caruaru, Brazil in 1996, had already suggested that MCs could pass the blood–brain barrier and induce neurotoxic symptoms [4]. Quantitative records of the behavior of two fish species, *Danio rerio* Hamilton and *Leucaspius delineatus* Heckel, showed that even exposure to low (0.5 µg·L^−1^) concentrations of MC-LR significantly modulated the swimming performance of these fish suggesting that there could possibly be a neurotoxic action [44]. This assumption gets support from biochemical and histological studies. For instance, Fischer *et al.* [45] showed that members of the organic anion transporting polypeptides were involved in the uptake of MC-LR and are expressed in both the liver and brain of the clawed frog (*Xenopus laevis*), which implies that the brain may be one of the potential targets of MC-LR. Analyzing three South American fish species for their tissue distribution of MC-RR, Cazenave, *et al.* [46] found traces of MC in the brains of *Jenynsia multidentata* Jenyns, indicating that MC has the ability to cross the blood-brain barrier. These examples clearly illustrate the need for routine neurotoxic evaluations of *Microcystis* and most likely other cyanobacteria blooms by a cost-effective neurotoxicity assay. 

Very recently, L1 larvae of *C. elegans* were proven to respond to MC-LR at environmentally realistic concentrations [24]. MC-LR in this case impaired a central neurotransmitter. In particular, γ-aminobutyric acid (GABA)ergic neurons were lost in a concentration-dependent manner at concentrations ≥0.1 μg·L^−1^. GABA is the most abundant inhibitory neurotransmitter in vertebrates and invertebrates and functions in *C. elegans* as an excitatory as well as an inhibitory neurotransmitter [47].

Again, the demonstrated *C. elegans* assay used showed strong modifications that indicate neurotoxicity in five out of six variables, namely both locomotive variables (Figure 4C,F), pharyngeal pumping activity (Figure 5C), thermotaxis (Figure 8C) and mechanical sensory ability (Figure 9C). This outcome continues to confirm that the *C. elegans* assay is sensitive and robust enough to identify the neurotoxicity of novel untested chemicals. Similar to the ANA check, the implications for C. elegans in the environment based on the overall outcome of these assays may be equally adverse, although different traits were found to respond to the toxin. For instance, the MC-induced preference of environments with elevated temperatures may be risky, because *C. elegans* is thermo sensitive and stops reproduction at temperatures >25 °C [27].

The filtrate of a *M. aeruginosa* culture showed mild neurotoxic effects for some of the sensory functions in *C. elegans* indicating the general suitability of the presented assay. Even the *Microcystis* culture medium appears to contain neuroirritant compounds. However, autonomic functions were surprisingly stimulated during *Ma*cf exposure. This effect might be caused by *Microcystis* metabolites other than microcystin, such as brominated organic compounds [22] and will be addressed in a separate study. Nevertheless, even neurostimulation shows the applicability of the test described, since it indicates that the exposed chemicals target neurons.

## 6. Conclusions

Neurobehavioral testing is traditionally done using mammals, however there is an upward spiraling of costs in terms of time, resources, and number of animals. This inefficiency and the high costs associated with mammalian testing reduces the possibility to evaluate the neurotoxicity of various environmental chemicals and pollutants. *C. elegans*, as an emerging model in environmental and toxicological sciences, was chosen for establishing a neurobehavioral screening system according to the guideline used for mammalians. The presented data support the possible establishment of this neurobehavioral model. Every positive control achieved the expected effect in this model organism. Furthermore, the well-known neurotoxin ANA as well as the hepatotoxin MC-LR revealed adverse effects in five out of six life traits. Overall, *C. elegans* appears to have the potential to replace rodents as neurotoxicity model animals.
